# The Asylums Board and London Fevers

**Published:** 1896-10-10

**Authors:** 


### The Hospital

AN INSTITUTIONAL JOURNAL OF

The Medical Sciences and Hospital Administration,

WITH

AN EXTRA NURSING SUPPLEMENT.

VOL. XXI.—No. 524.] [OCT. 10, 1896.

### THE ASYLUMS BOARD AND LONDON FEVERS.

IN view of the reiterated complaints which arise from various quarters of the town in regard to the lack of accommodation in the hospitals of the Asylums Board, it may be well to recall to mind the fact that, so far as the history of previous years may be accepted as a guide to the future, we are as yet some weeks from the time when the greatest prevalence of scarlet fever generally occurs. Almost the same may be said in reference to diphtheria, the greatest prevalence of which generally slightly follows that of scarlet fever, so that in regard to neither of these diseases have we yet got to the worst. According to a statement made at a recent meeting of the Asylums Board by Sir Edwin Galsworthy, 520 more cases of scarlet fever had been notified during the preceding fortnight than during the corresponding period of last year. But, if we go back further still, we find that last year considerably more cases were notified in that time than at the corresponding time the year before, so that it is evident that the depression which naturally followed the considerable prevalence of the disease in 1893 is passing away, and that the scarlet fever curve is on the rise both in regard to its seasonal and its annual prevalence. The same is true also, and in a still more marked degree, in regard to diphtheria. We must, therefore, expect that for some time to come the demands for admission to the hospitals will increase, and the problem how these demands for admission are to be met is one of no small gravity.

Now, in regard to this, we wish to draw attention to a point which has been far too little considered, namely, the absurdly small accommodation which is provided for diphtheria, as compared with that at the disposal of those suffering from scarlet fever, notwithstanding the far greater gravity of the former disease.

It is not easy to understand on what principle the authorities have allotted their beds to these two diseases. Even in proportion to the number of cases notified, scarlet fever has far more than its due proportion of beds. But if we consider that nearly a quarter of all the cases of diphtheria which are notified die, while of the scarlet fever cases notified only a little over one in twenty ends fatally, it would seem reasonable that the resources of the Board should be far more largely devoted to combating the graver disease than is the case at present, and that, whatever may be done in regard to scarlet fever, every case of diphtheria which applies should be at once admitted. In the last annual report of the Asylums Board it is stated that the managers "in the middle of the year found their available accommodation exhausted, and were compelled to restrict the admissions of scarlet fever and diphtheria to the most urgent cases." Would it not have been more reasonable, instead of maintaining a fixed and artificial ratio between the number of beds available for each disease, for the managers, irrespective of any preconceived ideas, to have taken in the cases of diphtheria in preference to those suffering from scarlet fever?

Turning to the report of the Medical Officer of Health to the London County Council, in which matters of this sort are stated with greater definiteness than in the statistics issued by the Asylums Board, we find that in 1894, the last year dealt with, 62·9 per cent. of the cases of scarlet fever notified were admitted to the Board's hospitals, whereas of the cases of diphtheria, the far graver disease, only 32·8 per cent. were admitted. There seems a dash of red tape about all this. If the intention is to admit the "most urgent" cases, clearly a case of scarlet fever would have to be at least four times as ill, if one may be excused the expression, as an average case is before it could claim precedence over even an average case of diphtheria; and yet, if one may judge by statements in official reports and by the complaints made by the public, scarlet cases are still taken in to the exclusion of diphtheria patients. To show still further the desirability of attending first to diphtheria, it should be mentioned that, notwithstanding the comparative mildness of scarlet fever, every case of that disease which is taken in occupies a bed for a far longer time than a case of diphtheria does, the average stay in hospital being seventy days for scarlet fever patients, while for diphtheria patients it is only forty-two days, so that the retention of accommodation for scarlet fever cases when diphtheria cases are waiting for admission is doubly extravagant of beds.

Of course the objection will be at once raised that to refuse cases of scarlet fever would be to let loose that disease on the metropolis. This, however, may be questioned. The great drop in the death-rate from scarlet fever in London took place when only quite an insignificant proportion of the cases were being isolated; while, as for recent years, the closing of the schools during the holidays seems to have a greater influence in breaking the usual autumnal rise of the disease than all the hospitals are able to effect. But, in any case, diphtheria is the worse enemy of the two. Moreover, this matter is outside the obligations of the hospital managers. Their duty clearly is to use their hospitals as places for the treatment of disease rather than the protection of the healthy, which is a matter for the sanitary authorities, and so far as treatment is concerned they certainly will do the greatest service by taking in the most urgent cases. But from a mere sanitary standpoint it would be easy to maintain that the complete isolation of one fatal and contagious malady, such as diphtheria is, would be a more useful proceeding than the partial isolation of two diseases and the devotion of the greater number of beds to that of the least fatality, which at the present time is the official system.

